# Supplementary material for: Hallucinations after Cardiac Surgery: A Prospective Observational Study
Source: Medicina (Kaunas). 2020 Mar 2;56(3):104. doi: 10.3390/medicina56030104 (PMC7143233; doi:10.3390/medicina56030104)
Supplement: Supplementary file 1 [file medicina-56-00104-s001.pdf]

Supplemental Material:

Table S1. Characteristics of visual and auditory hallucinations in the cardiac surgery group (n=201).

	Visual Hallucinations (N = 39)	Auditory Hallucinations (N = 9)
**Duration**		
Very brief, just an instant	4 (10.3)	4 (44.4)
A few seconds	8 (20.5)	0
A minute or a few minutes	11 (28.2)	1 (11.1)
10 minutes to an hour	4 (10,3)	1 (11.1)
Between one and several hours	6 (15.4)	2 (22.2)
(Almost) continually	5 (12.8)	0
Unknown	1 (2.6)	1 (11.1)
**Time of occurrence**		
No time pattern noticed	6 (15.4)	0
Only at night	9 (23.1)	4 (44.4)
Mostly during the evening	3 (7.7)	0
At the borders of sleep	5 (12.8)	1 (11.1)
Mostly during the day	8 (20.5)	1 (11.1)
Both during the day and night	5 (12.8)	1 (11.1)
Unknown	3 (7.7)	0
**Emotional valence**		
Never negative; the entire content is positive, useful or neutral	32 (82.1)	7 (77.8)
Occasional instances of negative content (< 10%)	2 (5.1)	1 (11.1)
Some of the content is negative	1 (2.6)	0
About half of the content is negative	0	0
The majority of the content is negative	1 (2.6)	0
Always, the entire content is negative	3 (7.7)	1 (11.1)
**Experienced distress**		
No discomfort, it does not affect me at all	30 (76.9)	8 (88.9)
Doubtful, perhaps a little discomfort	1 (2.6)	0
Some discomfort, it may affect my behavior or mood	3 (7.7)	0

Considerable discomfort, making me feel anxious, restless or depressed	1 (2.6)	0
A lot of discomfort, making me feel afraid, unsafe or depressed	3 (7.7)	1 (11.1)
Unknown	1 (2.6)	0
**Insight**		
Completely convinced that the hallucinations are not real	30 (76.9)	
Slight doubt whether they are real, most likely not real	1 (2.6)	
They are probably real, but alternative explanations may also be possible	1 (2.6)	
Strongly convinced that the hallucinations are real, only a little doubt	2 (5.1)	
Completely convinced that the hallucinations are real	4 (10.3)	
Unknown	1 (2.6)	
**Complexity (visual)**		
Simple forms like circles, flashes of light, dots or lines	9 (23.1)	
Patterns, i.e. checkerboards, diamond shaped objects, lattices or bricks	5 (12.8)	
(Distorted) faces or shadows	8 (20.5)	
Complex images: people and/or animals	5 (12.8)	
Complex images: inanimate objects (e.g. buildings, vehicles)	11 (28.2)	
Unknown	1 (2.6)	
**Complexity (auditory)**		
Only non-verbal auditory hallucinations, no voices		2 (22.2)
One single word		1 (11.1)
Several words, short phrases		0
Single sentences		3 (33.3)
Several sentences running together		2 (22.2)
Unknown		1 (11.1)

All values presented as N (%).

Table S2. Univariable logistic regression coefficients of potential risk factors for developing postoperative hallucinations (n=201).

	**Patients without Hallucinations N = 157**	**Patients with Hallucinations N = 44**	**Bèta**	**OR**	**Confidence Interval**	***P*-value**
Age, years, median (IQR)	64 (57 to 72)	69 (62 to 78)	0.047	1.048	1.01 to 1.09	**0.009**
Male sex (male=0)	113 (72)	33 (75)	-1.155	0.856	0.40 to 1.84	0.691
Diabetes managed with insulin	15 (9.6)	1 (2.3)	-0.513	0.22	0.03 to 1.72	0.148
Presence of coronary artery disease	100 (63.7)	29 (65.9)	0.097	1.102	0.55 to 2.23	0.787
Presence of valvular disease	91 (58)	25 (56.8)	-0.047	0.954	0.49 to 1.88	0.892
Presence of extracardiac arteriopathy	15 (9.6)	3 (6.8)	-0.367	0.693	0.19 to 2.51	0.576
History of cardiac surgery	12 (7.6)	1 (2.3)	-1.269	0.281	0.04 to 2.22	0.229
Chronic lung disease	14 (8.9)	5 (11.4)	0.270	1.31	0.44 to 3.86	0.625
Benzodiazepine premedication	135 (86)	29 (65.9)	-1.155	0.315	0.15 to 0.68	**0.003**
Duration of cardiopulmonary bypass, minutes, mean (SD)	115 (54)	133 (78)	0.005	1.005	1.00 to 1.01	**0.075**

Postoperative transfusion of blood products		42 (26.8)	19 (43.2)	0.733	2.081	1.04 to 4.16	**0.038**
Cumulative postoperative benzodiazepine dose, IV midazolam equivalent (mg), median, IQR		3 (0-10)	6 (0-14)	-0.002	0.998	0.99 to 1.01	0.773
Cumulative postoperative opoid dose, IV morphine equivalent (mg), median, IQR		12 (5-26)	18 (6-32)	0.007	1.007	0.99 to 1.02	0.371
Postoperative delirium		16 (10.2)	10 (22.7)	0.952	2.592	1.08 to 6.21	**0.033**
				Wald statistic	OR	Confidence interval	P-value
Renal impairment							
	No (GFR > 85)*	92 (58.6)	23 (52.3)	0.766			0.682
	Moderate (GFR 51-85)	53 (33.8)	18 (40.9)	0.729	1.358	0.67 to 2.75	0.393
	Severe (GFR <50) or on dialysis	12 (7.6)	3 (6.8)	0	1	0.26 to 3.84	1
Symptom severity NYHA / CCS- class							
	I*	38 (24.2)	4 (9.1)	5.667			0.129

	II	66 (42)	24 (54.5)	4.613	3.455	1.12 to 10.71	0.032
	III	36 (22.9)	13 (29.5)	3.988	3.431	1.02 to 11.51	0.046
	IV	17 (10.8)	3 (6.8)	0.399	1.676	0.34 to 8.32	0.527
Left ventricular ejection fraction							
	Good (>50%)*	98 (62.4)	27 (61.4)	0.461			0.794
	Moderate (31-50%)	44 (28)	14 (31.8)	0.147	1.155	0.55 to 2.41	0.702
	Poor (<30%)	15 (9.6)	3 (6.8)	0.229	0.726	0.20 to 2.69	0.632

Values represent n (%), unless indicated otherwise. * reference category.
